# A remarkable case of thyrotoxicosis initially caused by graves’ disease followed by a probable TSHoma – a case report

**DOI:** 10.1186/s12902-020-00611-7

**Published:** 2020-08-27

**Authors:** Mark Quinn, Waiel Bashari, Diarmuid Smith, Mark Gurnell, Amar Agha

**Affiliations:** 1 Department of Diabetes and Endocrinology, Beaumont Hospital and the RCSI, Dublin, Ireland; 2 Wellcome Trust-MRC Institute of Metabolic Science, University of Cambridge and National Institute for Health Research Cambridge Biomedical Research Centre, Addenbrooke’s Hospital, Cambridge, CB2 0QQ UK

**Keywords:** Graves’ disease, TSH-secreting pituitary adenoma, TSHoma, Thyrotropinoma, Coexistent primary and secondary hyperthyroidism

## Abstract

**Background:**

Graves’ disease is the commonest cause of thyrotoxicosis whilst thyrotropin (TSH)-producing pituitary adenomas (thyrotropinomas, TSHomas) are very rare and account for just 1–2% of all pituitary adenomas. Coexistence of a TSHoma and Graves’ disease has been very rarely reported. Here, we report a case of a patient whose initial presentation with primary thyrotoxicosis due to Graves’ disease, was subsequently followed by a relapse of thyrotoxicosis due to a probable TSHoma.

**Case:**

A sixty-eight year old woman was referred to our department with classical features of thyrotoxicosis. Initial biochemistry confirmed hyperthyroxinaemia [free thyroxine (fT4) 20.4 pmol/L (reference range 7.0–16.0)] and a suppressed TSH [< 0.02mIU/L (0.50–4.20)]. A technetium pertechnetate uptake scan was consistent with Graves’ Disease. She was treated with carbimazole for 18 months and remained clinically and biochemically euthyroid. After stopping carbimazole her fT4 started to rise but TSH remained normal. Laboratory assay interference was excluded. A TRH stimulation test demonstrated a flat TSH response and pituitary MRI revealed a microadenoma. Remaining pituitary hormones were in the normal range other than a slightly raised IGF-1. An ^11^C-methionine PET/CT scan coregistered with volumetric MRI (Met-PET-MRI^CR^) demonstrated high tracer uptake in the left lateral sella region suggestive of a functioning adenoma. The patient declined surgery and was unable to tolerate cabergoline or octreotide. Thereafter, she has elected to pursue a conservative approach with periodic surveillance.

**Conclusion:**

This is a very unusual case of thyrotoxicosis caused by two different processes occurring in the same patient. It highlights the importance of considering dual pathology when previously concordant thyroid function tests become discordant. It also highlights a potential role of Met-PET-MRI^CR^ in the localisation of functioning pituitary tumours.

## Background

Graves’ disease is the most common cause of thyrotoxicosis [1]. It occurs when autoantibodies (TRAb) bind to thyroid stimulating hormone (TSH) receptors driving unregulated production of thyroid hormones [triiodothyronine (T3) and thyroxine (T4)] that is independent of pituitary TSH. The diagnosis is based on the presence of typical features of thyrotoxicosis (± pathognomonic signs, e.g. dysthyroid eye disease), raised free thyroid hormones (fT3 and fT4) with suppressed TSH (traditionally < 0.1 mU/L), high TRAb titres and/or characteristic increased diffuse uptake on thyroid scintigraphy (using iodine or technetium).

In contrast, TSH producing pituitary adenomas (TSHomas, thyrotropinomas) are a much rarer cause of thyrotoxicosis [2]. They are associated with a biochemical pattern of central/secondary hyperthyroidism (elevated T3/T4 with a non-suppressed TSH), which is distinct from that of Graves’ disease and other causes of primary hyperthyroidism. The diagnosis is often challenging, reflecting significant variation in clinical manifestations, difficulty in confirming genuine hyperthyroxinaemia with non-suppressed TSH, and the increasing recognition that a significant proportion of TSHomas are microadenomas which are not always readily visualized on magnetic resonance imaging (MRI) [3]. A 2014 retrospective study of all histopathologically proven TSHomas over a 10 year period from a single centre confirmed this wide spectrum of clinical presentations with 34% presenting with visual field disturbance, 25% with thyrotoxicosis, 13% with secondary amenorrhoea and 9% with headaches [4].

The distinction between primary and secondary hyperthyroidism is important as the treatment is different for each condition. For example, treating thyrotoxicosis secondary to a TSHoma with antithyroid medications, radioactive iodine or thyroid surgery, as one would with primary thyroid disorders, can reduce the negative feedback on a TSHoma and promote tumour growth and potentially worsen thyrotoxicosis [4].

There are, however, a small number of case reports of both Graves’ disease and a TSHoma co-existing [5–13] This combination poses a number of diagnostic and therapeutic challenges, including localising microadenomas when MRI is indeterminate. In recent years functional imaging with 11-C-methionine PET-CT for pituitary adenomas has been employed to help diagnose and accurately localise functional pituitary tumours [14–16]

Here, we describe a patient initially diagnosed with primary thyrotoxicosis due to Graves’ disease, who subsequently developed secondary thyrotoxicosis from a probable TSHoma. We also report the use of ^11^C-methionine positron emission tomography to aid the localisation of this condition.

## Case presentation

A 68-year-old lady was originally referred to our endocrine service in 2011 with a diagnosis of thyrotoxicosis. Her thyroid function tests panel are shown in Table 1. She was asymptomatic and appeared clinically euthyroid with a resting heart rate of 68 beats per minute and an unremarkable head and neck exam. Her baseline ECG was normal. She had no known family history of any thyroid disorders.

**Table 1:** Thyroid Function Tests & Endocrine Treatment

Date	Endocrine Treatment	Free T4 (7.0–16.0 pmol/L)	TSH (0.5–4.2 mU/L)
July 2011	CBZ commenced	20.4	< 0.02
November 2011	CBZ 5 mg/day	9.9	3.11
November 2012	CBZ 5 mg/day	14.6	1.65
January 2013	CBZ discontinued		
March 2013	nil	17.7	0.82
May 2013	nil	20.4	0.63
July 2014	nil	21.5	0.67
October 2015	nil	20.1	0.77

Serial thyroid function tests prior to, during, and following discontinuation of antithyroid drug therapy

*Key*: *CBZ* carbimazole, *T4* thyroxine, *TSH* thyroid stimulating hormone

**Fig. 1:**
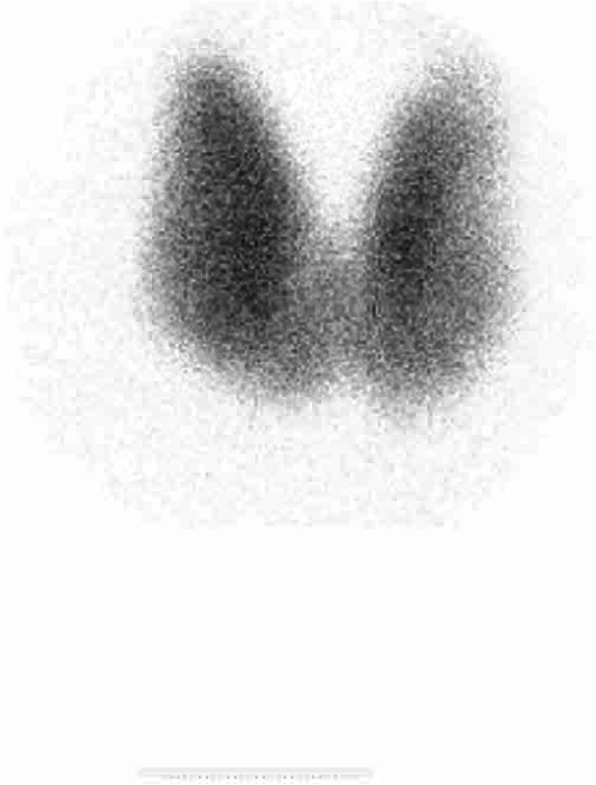
Thyroid uptake scan. Thyroid uptake scan (technetium-99 m pertechnetate) demonstrating homogenous tracer uptake in both lobes

A technetium-99 m pertechnetate thyroid uptake scan demonstrated homogenous diffuse tracer uptake in both lobes in keeping with a diagnosis of Graves’ disease (Fig. 1.). Sex hormone binding globulin was 92 nmol/l (27–128).

She was started on carbimazole and remained clinically and biochemically euthyroid for the following 18 months (Table 1).

Carbimazole was stopped in January 2013 and the patient was followed in our clinic with serial thyroid function tests. She remained asymptomatic but her blood tests over the next 2 years demonstrated a discordant pattern – a persistently elevated free T4 with a TSH level inappropriately in the normal range (Table 1). She was clinically euthyroid without a goitre.

The same pattern was confirmed on several different laboratory platforms and assay interference was formally excluded. TFTs on the 2 step Delfia platform showed a free T4 of 26.8 pmol/L (9.0–20), a free T3 of 8.5 nmol/L (3.0–7.5) and a TSH of 0.74 mU/L (0.4–4.0) while TFTs on the 1 step Centaur platform showed a free T4 of 23.9 pmol/L (10.0–19.8), a free T3 of 6.7 pmol/L (3.5–6.5) and a TSH of 0.76 mU/L (0.35–5.50). The alpha subunit was in the normal range [0.9 IU/L (RR < 1.0)], but a thyrotropin releasing hormone (TRH) stimulation test demonstrated a flat TSH response (TSH 0.73 / 0.72 / 0.70 mU/L at 0, 20 and 60 min respectively). A pituitary blood profile was unremarkable aside from a mildly elevated serum insulin-like growth factor 1 level (1.2 × upper limit of normal) (Table 2). An oral glucose tolerance test (OGTT) was performed as a growth hormone suppression test. This showed borderline growth hormone suppression with a nadir of 0.43 ng/mL (Table 3). The patient had no clinical features of acromegaly. A T3 suppression test was considered but following discussion with the patient she decided against this.

**Table 2:** Pituitary Blood Profile

	08/08/2016	Ref. range
AM Cortisol (nmol/L)	373	185–624
FSH (mIU/mL)	98.5	30–120
LH (mIU/mL)	36.2	15–62
Basal Growth Hormone (ng/mL)	0.96	
IGF-1 (ng/mL)	**201**	37–166
Prolactin (mIU/L)	247	58–416

Repeat pituitary blood profile

*Key*: *FSH* follicle-stimulating hormone, *LH* luteinizing hormone, *IGF-1* insulin-like growth factor 1

**Table 3:** Oral Glucose Tolerance Test

Oral Glucose Tolerance Test		
Time (minutes)	Blood Glucose (mmol/L)	Growth Hormone (ng/ml)
0	5.2	7.07
30	9.0	1.63
60	9.0	0.78
90	5.2	0.57
120	4.5	0.43
150		0.45

A summary of the existing case reports of patients with both Graves’ disease and a TSHoma

*GD* Graves’ Disease, *CBZ* Carbimazole, *TSS* Transsphenoidal surgery, *PTU* Propylthiouracil, *MMI* Methimazole

**Fig. 2:**
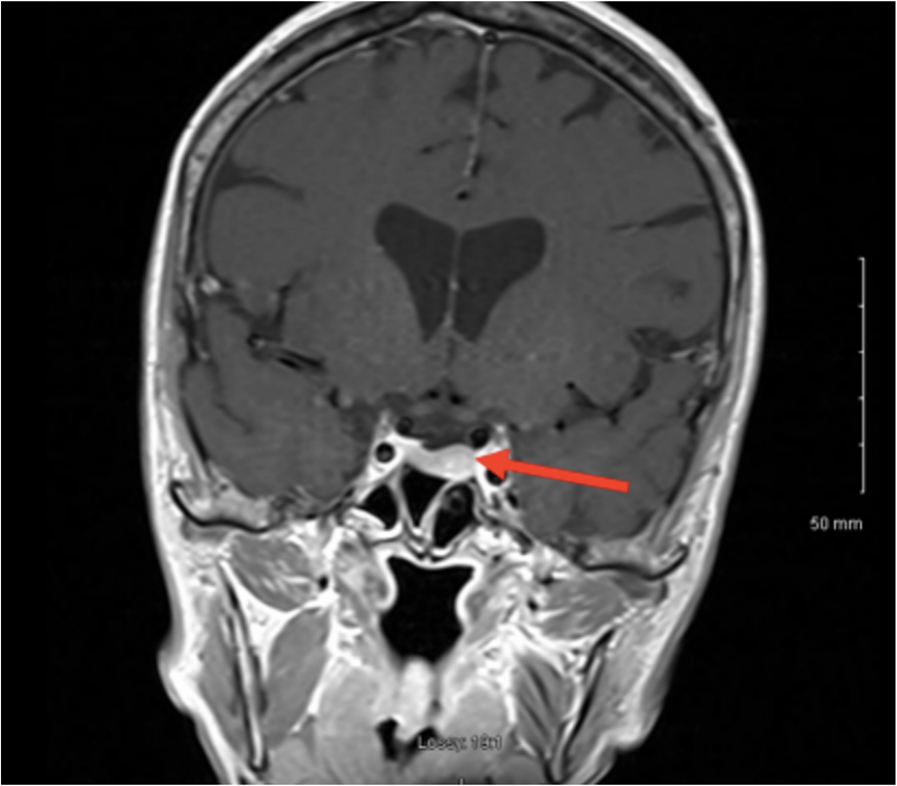
MRI Pituitary. MRI Pituitary showing enlargement of the left side of the pituitary - findings suspicious for a pituitary microadenoma

**Fig. 3:**
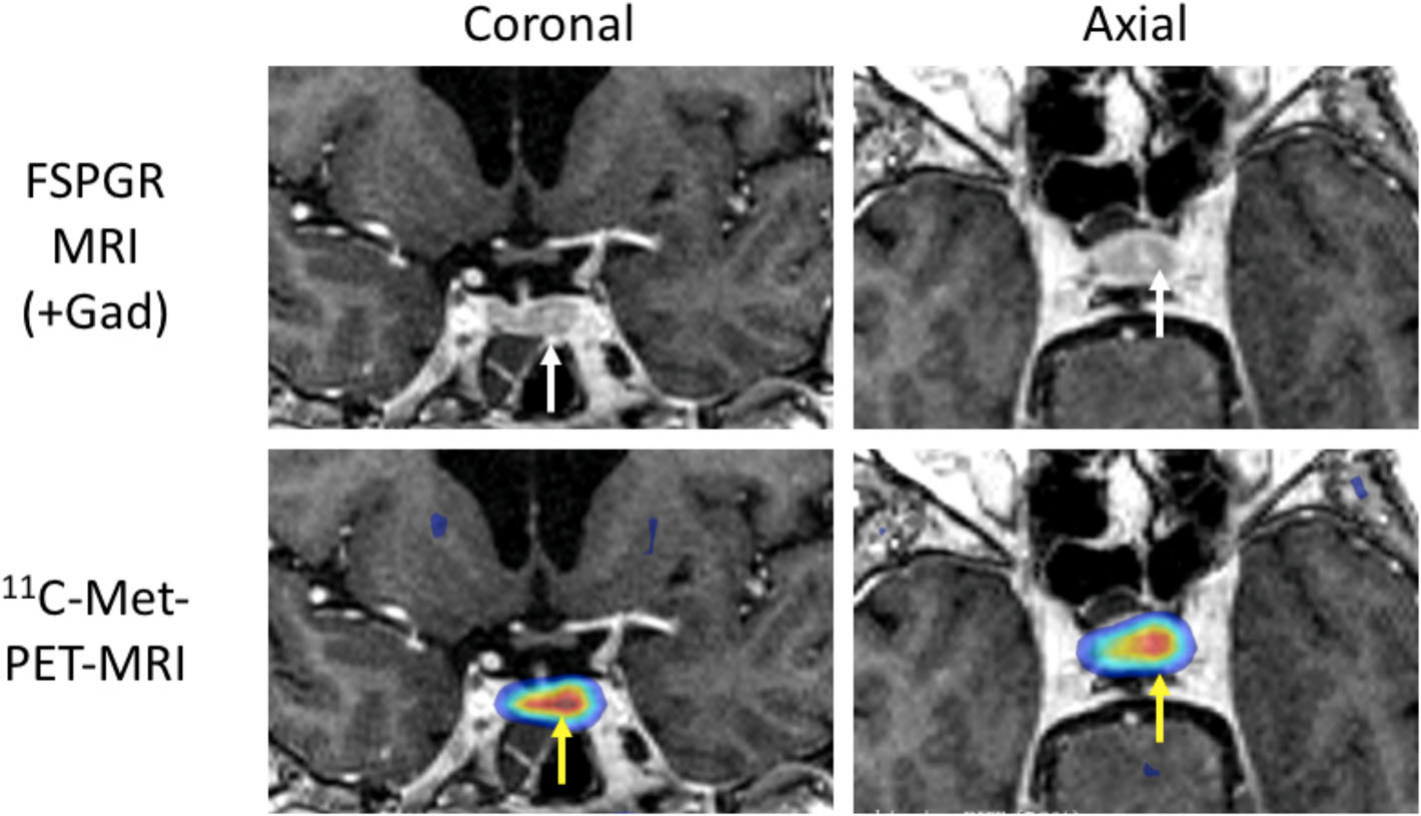
^11^C-methionine PET/CT coregistered with volumetric MRI. ^11^C-methionine PET/CT coregistered with volumetric MRI Cornonal and axial views showing a focus of increased tracer uptake in the left side of the sella (yellow arrows) corresponding to the site of a possible microadenoma on MRI (white arrows)

**Fig. 4:**
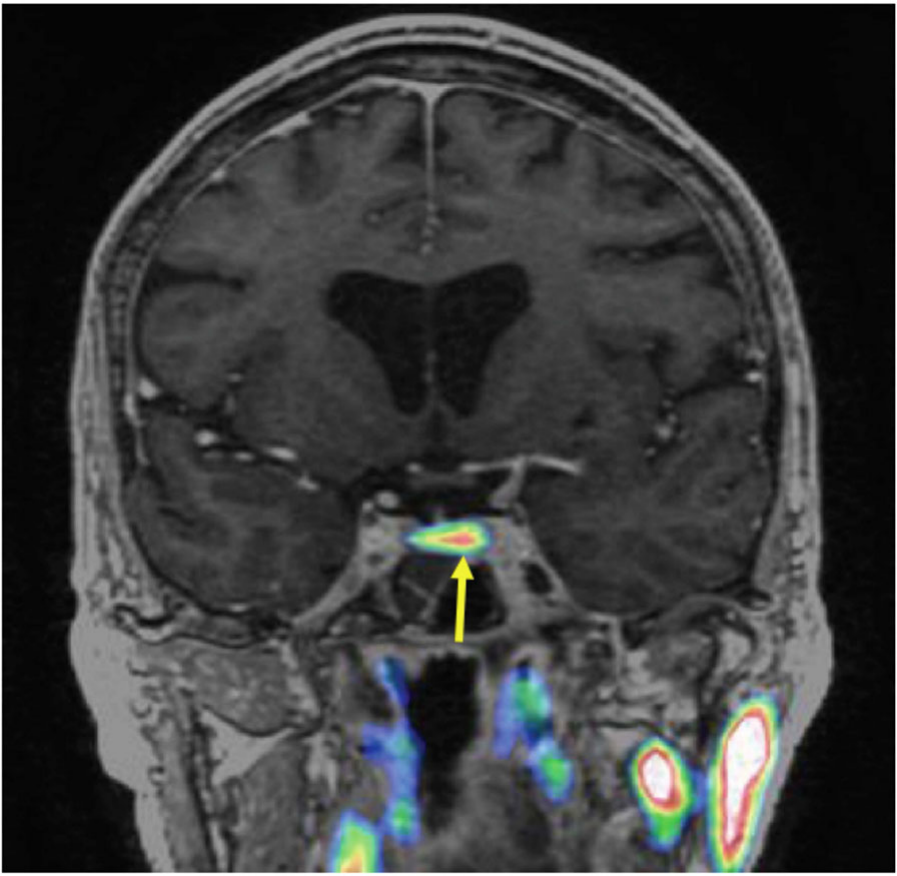
^11^C-methionine PET/CT coregistered with volumetric MRI. Coronal view

A pituitary MRI scan showed asymmetric enlargement of the gland, raising the possibility of a left-sided pituitary microadenoma (Fig. 2). An ^11^C-methionine PET/CT scan was performed and coregistered with a volumetric [fast spoiled gradient recall (FSPGR) MRI scan (Met-PET-MRI^CR^)]. This demonstrated focal increased tracer uptake at the site of the suspected microadenoma (Figs. 3 and 4). Her biochemical and radiological findings were therefore consistent with the diagnosis of a TSH-secreting pituitary adenoma. The slightly raised IGF-1 and borderline suppression of GH on OGTT also raised the possibility of GH co-secretion. The patient was tried on a somatostatin analogue (SSA) first then a dopamine agonist but did not tolerate either (due to gastrointestinal side-effects). She declined pituitary surgery. She is currently managed with a beta-adrenergic blocker and is clinically euthyroid.

## Discussion and conclusion

This is an unusual case of a patient with dual pathology: primary thyrotoxicosis due to Graves’ disease (GD) and secondary hyperthyroidism due to a probable TSHoma. Careful interpretation of laboratory and radiological findings allowed both conditions to be identified in a timely manner, and guided choice of therapy.

In cases of primary thyrotoxicosis (high free T4 and suppressed TSH) current guidelines suggest measuring anti-TSH receptor antibody (TRab) levels at an early stage [17]. TRab levels are positive in 90 [18]–99% [19] of cases of GD depending on the generation of assay used. In contrast, thyroid peroxidase (TPO) antibodies are only positive in 75–80% of cases of GD [20]. Thyroid scintigraphy and/or ultrasound may provide additional diagnostic information as the most common causes of primary hyperthyroidism, Graves’ disease and toxic multinodular goitre, have characteristic features on imaging [21].

For cases of suspected central (secondary) hyperthyroidism it is essential to exclude laboratory assay interference before proceeding with further investigations. Circulating heterophilic antibodies or human anti-animal immunoglobulins may cause spurious TSH results. Several approaches can be employed to detect such interference. At the simplest level, the demonstration of discordant results when TSH is measured using two different assay platforms is reasonable evidence of assay interference. However, other techniques [e.g. TSH measurement following serial dilution or polyethylene glycol (PEG) precipitation] provide more robust assessments of TSH integrity [22].

Similarly, free thyroid hormone levels may be artifactually raised and consideration should also be given to excluding causes of FT4 (±FT3) assay interference.

Once genuine hyperthyroxinaemia with non-suppressed TSH has been confirmed, investigations are targeted towards differentiating between a TSH-secreting pituitary adenoma and thyroid hormone resistance due to a loss-of-function mutation in the human *THRB* gene. Classically, TSHomas exhibit a blunted response to TRH stimulation, elevated sex hormone binding globulin (SHBG), raised alpha subunit (ASU), and reduction in thyroid hormone levels in response to depot SSA therapy. However, these findings are not universal. Our patient demonstrated no change in TSH following TRH injection, but ASU was within normal limits. She was unable to tolerate SSA.

Pituitary imaging with T1- and T2-weighted MRI remains the gold standard for identifying pituitary adenomas [23]. However, interpretation of pituitary MRI scans can be complicated by the high rate of pituitary incidentalomas in the general population. One large study found pituitary adenomas (mostly microadenomas) in 10.6% of subjects at autopsy [23]. This was in a group of patients who were not suspected of having pituitary disease while they were alive.

Whilst TSHomas are very rare they co-secrete other pituitary hormones in a high proportion of cases. This most commonly involves co-secretion of growth hormone (16% of cases) and prolactin (10%) [24]. In our case the elevated levels of IGF-1 raised the possibility of growth hormone hypersecretion. There were no signs of acromegaly and an oral glucose tolerance test (OGTT) demonstrated growth hormone suppression to 0.43 ng/mL. While this could possibly represent very low grade autonomous growth hormone secretion we felt this was unlikely given the lack of any clinical features of acromegaly. In a case with borderline results it is also worth noting that GH nadir levels are often higher in females than in males [25].

In recent years, functional pituitary imaging has been proposed as a useful tool for identifying the site(s) of a pituitary adenoma in patients with inconclusive MRI findings [26]. ^11^Carbon-methionine is an amino acid based PET tracer which, unlike the glucose-based tracer ^18^F-fluorodeoxyglucose, is preferentially taken up by normal pituitary tissue [27] with relatively low uptake by background brain tissue. Coregistration of ^11^C-Methionine PET/CT and volumetric (e.g FSPGR) MRI may be superior to MRI alone in localizing some pituitary microadenomas [16, 28, 29]

Inferior petrosal sinus sampling (IPSS) is often considered the gold standard investigation for the diagnosis of functional pituitary tumours. This is an invasive test that requires significant technical expertise. While more data is needed on the utility of Met-PET-MRI^CR^ there is some evidence to suggest it is a highly sensitive non-invasive test that could be considered as an alternative to IPSS [30]. In our case, although MRI raised suspicion of a left-side abnormality, no definite adenoma was seen. However, Met-PET-MRI^CR^ confirmed focal tracer uptake corresponding to this site.

IPSS and a pituitary biopsy may have been useful investigations to confirm the diagnosis but they need to be considered in the clinical context. In this case they were both felt to be unnecessarily invasive in an asymptomatic patient.

### Literature review

Whilst the dual diagnosis of primary and central thyrotoxicosis in the same patient is recognised to be exceedingly rare, some case reports of this pattern have been published in recent years (Table 4). The majority of these cases were in females (80%) all aged between 25 and 53 years old. Interestingly the dual diagnosis was confirmed within 3 years of the original diagnosis in all cases. This has led to a number of theories in the literature suggesting that treatment of the original condition may in fact promote the development of the subsequent condition. In the cases where Graves’ disease was diagnosed initially (40%) one hypothesis is that treatment with antithyroid medications may promote the growth of a TSHoma, due to the positive feedback system, so accelerating the presentation [6]. Conversely a number of theories have been proposed to explain why Graves’s disease may present following treatment of a TSHoma. Kageyama et al. demonstrated that anti-thyrotropin receptor antibody levels were significantly elevated post removal of a TSHoma in a 21 year old lady [31]. The pathogenesis of this is not fully understood but it has been suggested it is due to an increase in Fas-antigen mediated apoptosis of thyrocytes [32] and upregulation of various cell surface markers implicated in auto-immune disease (intercellular adhesion molecule-1, major histocompatibility complex II) [9] in response to a sudden fall in TSH levels.

**Table 4:** Literature Review

Author	Where	Year	Diagnosed 1st	Time between diagnosis	Antibodies	Sex	Age	Treatment
Aria N [3]	Japan	2016	Simultaneous	NA	Positive	F	40	CBZ- > TSS
Okyucu K [4]	Turkey	2016	Simultaneous	NA	Positive	F	37	PTU - > Thyroidectomy- > TSS
Ogawa Y [5]	Japan	2013	GD	2 years	Positive	F	32	PTU- > TSS
Koriyama N [6]	Japan	2004	TSHoma	3 years	Negative	F	37	Octreotide- > TSS - > CBZ
Kamoi K [7]	Japan	1985	TSHoma	10 months	Positive	F	46	MMI- > TSS- > MMI
Kamoun M [8]	France	2014	GD	2 years	Positive	F	36	CBZ - > Thyroidectomy - > TSS
Sandler R [9]	US	1976	TSHoma	2 years	Negative	F	53	CBZ- > Pituitary radiotherapy - > PTU- > ^131^I
O’Donnell J [10]	N.Ireland	1973	TSHoma	2 months	Negative	M	25	CBZ - > Hypophysectomy - > CBZ
Lee MT [11]	Taiwan	2010	GD	2 years	Positive	M	27	CBZ
Lee MT [11]	Taiwan	2010	GD	6 months	Positive	F	28	PTU

Current guidelines for the treatment of a TSHoma recommend surgery as first line [33]. In our case the patient declined this approach. Second line therapy in the form of SSAs was then put in place. She did not tolerate this so cabergoline was tried. Again, she failed to tolerate this. Going forward we have decided to treat this lesion conservatively with a beta-adrenergic blocker especially as she was clinically euthyroid. Beta-blockers were continued to protect against the cardiac effects of her biochemical thyrotoxicosis. We also used Denosumab to treat her osteoporosis. She remains well on this regime and attends our department for regular follow up.

### Summary

This case highlights the need to consider a diagnosis of a TSHoma when faced with discordant thyroid function tests. It also highlights the specific diagnostic and therapeutic challenges associated with the metachronous diagnoses of primary and central thyrotoxicosis in the same patient. We have used this case to discuss the current diagnostic tools at our disposal for the investigation of thyrotoxicosis. In addition, this case highlights the utility of an ^11^C-methionine PET/CT scan coregistered with volumetric MRI in the diagnosis of functional endocrine tumours especially in cases of diagnostic uncertainty. The limitations of this case report include the lack of TSH receptor antibody levels, the lack of IPSS and the lack of a confirmatory histological diagnosis.

## Data Availability

NA

## Abbreviations

TSH: Thyroid Stimulating Hormone
TRH: Thyrotropin Releasing Hormone
TRAb: TSH Receptor Antibody
TPO: Thyroid Peroxidase
GD: Graves’ Disease
MRI: Magnetic Resonance Imaging
IGF-1: Insulin-Like Growth Factor-1
PET-CT: Positron Emission Tomography-Computed Tomography
SPGR: Spoiled Gradient Recalled
TFT: Thyroid Function Test
FSH: Follicle-Stimulating Hormone
LH: Luteinising Hormone
CBZ: Carbimazole
TSS: Transsphenoidal surgery
PTU: Propylthiouracil
MMI: Methimazole
